# Chemical Components and Hepatoprotective Mechanism of Xwak Granule in Mice Treated with Acute Alcohol

**DOI:** 10.1155/2020/8538474

**Published:** 2020-09-28

**Authors:** Li Chen, Liu Liu, Rahima Abdulla, Xirali Tursun, Xuelei Xin, Haji Akber Aisa

**Affiliations:** ^1^Key Laboratory of Chemistry of Plant Resources in Arid Regions, State Key Laboratory Basis of Xinjiang Indigenous Medicinal Plants Resource Utilization, Xinjiang Technical Institute of Physics and Chemistry, Chinese Academy of Sciences, Urumqi 830011, China; ^2^University of Chinese Academy of Sciences, Beijing 100049, China

## Abstract

**Objective:**

To evaluate the hepatoprotective mechanism of Xwak granule (Xwak) in treatment of mice with alcoholic liver injury via activating ERK/NF-*κ*B and Nrf/HO-1 signaling pathways.

**Methods:**

The chemical composition of Xwak was tested by liquid chromatography coupled with mass spectrometry (LC-MS). Herein, 1,1-diphenyl-2-picrylhydrazyl (DPPH) scavenging assay and 2,2-azino-bis (3-ethylbenzothiazoline-6-sulphonic acid (ABTS) radical tests were performed *in vitro*. The hepatoprotective effect of Xwak was assessed at different concentrations (1.5, 3, and 6 g/kg) in a mouse model of alcoholic liver injury.

**Results:**

Totally, 48 compounds, including 16 flavonoids, 8 tannins, 9 chlorogenic acids, and 15 other compounds, were identified from Xwak. Xwak showed to have a satisfactory antioxidant activity *in vitro*. In a group of Xwak-treated mice, the serum levels of alanine transaminase (ALT), aspartate transaminase (AST), and alkaline phosphatase (ALP) were decreased compared with a group of the mouse model of alcoholic liver injury. In addition, the levels of antioxidant enzymes, such as glutathione peroxidase (GSH-PX), total superoxide dismutase (T-SOD), and catalase (CAT), were noticeably increased and the levels of malondialdehyde (MDA), tumor necrosis factor-*α* (TNF-*α*), transforming growth factor-*β* (TGF-*β*), and interleukin-6 (IL-6) were markedly reduced in the liver of mice. The state of oxidative stress in the mouse model of alcoholic liver injury was improved after treatment with Xwak. The improvement of inflammation-mediated disruption may conducive to the Xwak activity in the control of liver injury. The signals of p-ERK1/2, p-NF-*κ*B, COX-2, iNOS, CYP2E1, Nrf, and HO-1 were significantly induced in the liver of mice after treatment with Xwak.

**Conclusions:**

The abovementioned findings indicated that the hepatoprotective mechanism of Xwak could be achieved by activating ERK/NF-*κ*B and Nrf/HO-1 signaling pathways to alleviate oxidative stress and inflammatory.

## 1. Introduction

Alcoholic liver disease (ALD) is a term that encompasses the liver manifestations of alcohol overconsumption, including fatty liver, alcoholic hepatitis, and chronic hepatitis with liver fibrosis or cirrhosis [1]. The initial stage of ALD is liver damage caused by excessive consumption of alcohol, and in case of late treatment, it may further develop from alcoholic hepatitis to alcoholic fibrosis and finally deteriorate to alcoholic cirrhosis, and even hepatocellular carcinoma. ALD cause substantial morbidity and mortality, with around 500,000 deaths/year worldwide, and it adversely affects the quality of life of patients and their family members [2–4]. Prevention and treatment of ALD have become increasingly serious public health concerns worldwide. The underlying molecular mechanisms of ALD progression are still in progress, and previous research studies revealed that important factors influencing the progression of ALD are oxidative stress, lipid peroxidation, inflammation, and formation of toxic by-products [5–7]. In addition, oxidative stress plays a pivotal role in the occurrence and development of ALD. Dysregulated cytokine metabolism is another characteristic of ALD [8, 9]. Therefore, it is highly urgent to find out new natural medicines to improve the progression of ALD according to the pathogenesis factors.

Natural plant extracts have significantly attracted scholars' attention due to their advantages of low toxicity, easy absorption, and multitarget action [10–12], while further effective natural medicines for ALD need to be explored. Xwak is a classic folk prescription, originating from national medical master, with a long history of clinical application in Uygur Medical Hospital, Xinjiang, China. Xwak has been registered in National Intellectual Property Administration (Beijing, China). It is mainly used in the treatment of liver diseases in ethnic medicine. Xwak is composed of six traditional Chinese herbs, including *Artemisia rupestris* L., *Artemisia capillaris* Thunb., *Cichorium glandulosum* Boiss. et Huet., *Rosa rugosa* Thunb., *Rheum palmatum* L., and *Glycyrrhiza glabra* L. These herbs contain anti-inflammatory effects, improve immune responses, and protect the liver [13–21]. We found that the main chemical components in Xwak are flavonoids, tannins, chlorogenic acid and phenolic acid, etc., and these components have been reported to possess protective functions against liver diseases [10, 22–26]. However, the mechanism of hepatoprotection of Xwak in alcoholic liver injury has still remained elusive. In the present study, we analyzed the chemical components of Xwak and evaluated the hepatoprotective mechanism of Xwak in mice with alcoholic liver injury.

## 2. Materials and Methods

### 2.1. Materials and Reagents

Herein, 2,2-azino-bis(3-ethylbenzothiazoline-6-sulphonic acid) (ABTS) and 1,1-diphenyl-2-picrylhydrazyl (DPPH) were purchased from Sigma-Aldrich Corporation (St. Louis, MO, USA). Silybin was obtained from Tianjin Tianshili Shengte Pharmaceutical Co., Ltd. (Tianjin, China). Kits required for measuring the levels of aspartate aminotransferase (AST), alanine aminotransferase (ALT), glutathione peroxidase (GSH-PX), total superoxide dismutase (T-SOD), catalase (CAT), malondialdehyde (MDA), lactate dehydrogenase (LDH), total cholesterol (TC), and triglyceride (TG) were purchased from Nanjing Jiancheng Bioengineering Institute (Nanjing, China). The alkaline phosphatase (ALP) was purchased from Shenzhen Mindray Biomedical Electronics Co., Ltd. The nitric oxide (NO) assay kit was brought from Beyotime Biotechnology (Shanghai, China). The enzyme-linked immunosorbent assay (ELISA) kits required for measuring the levels of interleukin-6 (IL-6), tumor necrosis factor-*α* (TNF-*α*), and transforming growth factor-*β* (TGF-*β*) were purchased from MultiSciences (Hangzhou, China). The BCA protein assay kit was provided by Thermo Fisher Scientific (Waltham, MA, USA). The antibodies against p-ERK1/2, ERK1/2, p-NF-*κ*B, nuclear factor-kappa B (NF-*κ*B), COX-2, inducible NO synthase (iNOS), GAPDH, and *β*-actin were purchased from Cell Signaling Technology (Danvers, MA, USA). The antibody against cytochrome P450 2E1 (CYP2E1) was purchased from Millipore (Burlington, MA, USA). The antibodies against Nrf2 and HO-1 were purchased from Abcam (Cambridge, UK). The secondary antibody was provided by Boster Biological Technology Co., Ltd. (Wuhan, China). ECL Plus™ was obtained from GE Healthcare (Chicago, IL, USA).

### 2.2. Plant Material and Preparation

Herein, *Artemisia rupestris* L., *Artemisia capillaris* Thunb., *Cichorium glandulosum* Boiss.et Huet., *Rosa rugosa* Thunb., *Rheum palmatum* L., and *Glycyrrhiza glabra* L. were collected from the Traditional Uygur Medicine Hospital (Xinjiang, China). These herbs were identified by Anwar Talip, the director of the pharmaceutical department. Voucher specimens were deposited in Xinjiang Technical Institute of Physics and Chemistry, Chinese Academy of Sciences (Xinjiang, China; voucher nos. *Artemisia rupestris* L. WY01223; *Artemisia capillaris* Thunb. WY02656; *Cichorium glandulosum* Boiss.et Huet. WY02310; *Rosa rugosa* Thunb. WY02101; *Rheum palmatum* L. WY02658; *Glycyrrhiza glabra* L. WY02657). The six medicinal materials were washed, dried, and crushed. According to the extraction method of the Xwak patent, 16 times of the amount of water was added and extracted twice, 2.5 h each time. The combined extract was concentrated under reduced pressure and then dried at 60°C, crushed and sifted to obtain drug substance.

### 2.3. Ultrahigh-Performance Liquid Chromatography Coupled with Mass Spectrometry (UHPLC-Q-Orbitrap-MS) Analysis

#### 2.3.1. Liquid Chromatography

UHPLC analyses were conducted using the UltiMate 3000 Rapid Separation LC (RSLC) system (Thermo Scientific Dionex, Waltham, MA, USA). The chromatographic separation column was InertSustain C18 (4.6 × 250 mm, 5 *μ*m), in addition to gradient elution with a mobile phase consisting of 0.2% formic acid in water (solvent A) and acetonitrile (solvent B) using the following gradients: 0–8 min (4–7% B), 8–40 min (7–8% B), 40–45 min (8–10% B), 45–70 min (10–13% B), 70–120 min (13–16% B), 120–145 min (16–28% B), 145–150 min (28–35% B), and 150–170 min (35–50% B); the flow rate was 1.0 mL/min, and volume of injection was 10 *μ*L.

#### 2.3.2. Mass Spectrometry

The MS was conducted with a Quadrupole-Orbitrap-HRMS (Thermo Fisher Scientific, Bremen, Germany). A high-sensitive heated electrospray ionization (HESI) probe was used to nebulize and ionize samples in both positive and negative ion modes. The MS acquisition was carried out in data-dependent acquisition (DDA) mode: the range of full-scan was from 100 to 1500 *m/z* at 70,000 FWHM at 200 *m/z*; MS/MS was set as 17,500 FWHM at 200 *m/z*. The MS source was set as follows: heat temperature, 350°C; capillary temperature, 300 °C; source voltage, 3.2 kV (positive) and −2.8 kV (negative); sheath gas flow, 40 arb; and auxiliary gas flow, 10 arb. The stepped normalized collision energy (NCE) was set to 20%, 40%, and 60%. The instrument was controlled by Xcalibur 4.0 (Thermo Fisher Scientific Inc., USA).

### 2.4. Antioxidant Activity *In Vitro*

The DPPH scavenging assay was performed as described previously with minor modification [27]. DPPH is weighed accurately and dissolved with ethanol to prepare a solution with the concentration of 2 mM, stored it in a refrigerator, diluted to the concentration of 0.2 mM DPPH solution before use. Next, 100 *μ*L sample with different concentrations was mixed with 100 *μ*L DPPH (0.2 mM) solution, and the reaction mixture was incubated in the dark for 30 min at room temperature. Optical density (OD) of 515 nm was measured thereafter.

The ABTS radical assay is an excellent tool for determining the antioxidative activity, in which the radical is quenched to form ABTS radical complex. The ABTS radical scavenging activity was calculated according to Liu et al.'s method [28]. The ABTS solution was prepared by mixing with 7 mM ABTS and 2.45 mM K_2_S_2_O_8_ and allowing the mixture to stand in the dark at room temperature for at least 12 h. The stock solution was diluted with ethanol to reach 0.68–0.72 absorbance at 734 nm before use. The reaction was conducted with 16 *μ*L sample and 184 *μ*L ABTS working solution, and the mixed solution was reacted in the dark for 5 min at room temperature. Then, OD of 734 nm was measured.

Vitamin C was positive control in the ABTS and DPPH tests. The scavenging ability was calculated according to the following formula:

(1) $$\begin{array}{c} \text{The scavenging ability}\left(\text{\%}\right)=\left[\frac{\left({\text{OD}}_{\text{control}}-{\text{OD}}_{\text{sample}}\right)}{\left({\text{OD}}_{\text{control}}-{\text{OD}}_{\text{blank}}\right)}\right]\times 100\text{\%,} \end{array}$$

where OD_sample_, OD_blank_, and OD_control_ represent the absorbance of the sample, the blank, and the control, respectively.

### 2.5. Animal Experiments

Five-week-old ICR male mice were purchased from the Experimental Animal Center of Xinjiang Medical University. The mice were housed in conventional cages under constant temperature and humidity with free access to food and water. All experimental procedures were approved by the Xinjiang Uygur Medicine Research Institute (approval no. SYXK 2016–0003).

The mice were allowed to acclimate for one week prior to experiments. Then, a total of 48 mice were randomly divided into 6 groups (*n* = 8 for each group) as follows: control (Cont), alcoholic liver injury model group (EtOH), positive control (SILY), low-dose of Xwak (XL), medium-dose of Xwak (XM), and high-dose of Xwak (XH). In the Cont and EtOH groups, mice were given the same volume of distilled water. In the SILY group, mice were orally treated with silybin (79.1 mg/kg). In the three dose-based groups, mice were orally administered with a low-dose (1.5 g/Kg), medium-dose (3 g/Kg), and high-dose (6 g/Kg) of Xwak for 4 weeks. Additionally, 1 h after the last administration, mice in the Cont group were intraperitoneally injected with the same volume of saline, and in the other groups, mice were intraperitoneally injected with 56% ethanol at 5 mL/kg to induce hepatic injury as reported previously [29, 30]. Then, the mice were killed 5 hours later, and the serum and liver were collected for the subsequent experiments.

### 2.6. Blood Biochemical Analysis

The mouse blood was collected at the end of experiment. Then, serum was obtained by centrifugation at 3000 rpm for 10 min. The serum levels of AST, ALT, and ALP in a mouse model of alcoholic liver injury were detected by 7100 Hitachi automatic biochemical analyzer (Hitachi, Tokyo, Japan).

### 2.7. Detection of Biochemical Markers in Liver Tissue

The liver tissue of mice was accurately weighed, and liver tissue homogenate in saline or phosphate-buffered saline (PBS) buffer was prepared to detect the biochemical markers. The protein concentration of liver tissue homogenate was detected by BCA kit. The levels of AST, ALT, LDH, NO, TC, TG, GSH-PX, T-SOD, CAT, MDA, IL-6, TNF-*α*, and TGF-*β* were detected according to the manufacturer's instructions of kits.

### 2.8. Western Blot Analysis

The liver tissues were homogenized in lysis buffer, and the supernatant was collected after centrifugation at 13000 rpm for 10 min. The protein concentration was assayed by BCA kit. Equal amount of protein from each sample was subjected to sodium dodecyl sulfate-polyacrylamide gel electrophoresis (SDS-PAGE), and then the protein was transferred onto a polyvinylidene fluoride (PVDF) membrane. The membrane was blocked with 5% milk buffer or 5% bovine serum albumin (BSA) buffer for 1 h. The primary antibodies against p-ERK1/2, ERK1/2, COX-2, p-NF-*κ*B, NF-*κ*B, iNOS, CYP2E1, Nrf2, HO-1, GAPDH, and *β*-actin were used to detect the corresponding signals overnight at 4°C. After that, the membranes were incubated with horseradish peroxidase (HRP)-conjugated secondary antibody for 1 h. The specific band was detected with the chemiluminescent reagent (ECL) by Bio-Rad gel imaging system (Bio-Rad Laboratories, Hercules, CA, USA).

### 2.9. Histological Evaluation

The fresh liver of mice was fixed with 10% neutral formalin solution, dehydrated (TP1020, LEICA, Germany), paraffin embedded (KD-BM/BLII, Zhejiang, China), sliced (RM2235, LEICA, Germany), and hematoxylin eosin (HE) stained for histopathological examination [31].

### 2.10. Statistical Analysis

The data were expressed as the mean ± standard deviation (SD). The statistical difference between the groups was calculated with one-way analysis of variance (ANOVA). The data were statistically analysed by GraphPad Prism 6.0 software (GraphPad Software Inc., La Jolla, CA, USA). *p* < 0.05 was considered statistically significant.

## 3. Results

### 3.1. LC-MS Analysis of Xwak

Totally, 48 compounds, including 16 flavonoids, 8 tannins, 9 chlorogenic acids, and 15 other compounds, were identified from Xwak by LC-MS with the help of retention time (*t*_*R*_), predicted formula and errors, fragmentation behavior, and the reference standards (Table 1).

### 3.2. Antioxidant Activity of Xwak *In Vitro*

DPPH and ABTS were used to detect the antioxidant activity of the Xwak. The change of color caused by the reaction between antioxidants and radical can be observed at a specific wavelength. The antioxidant activity of Xwak was shown as the half maximal inhibitory concentration (IC_50_). In DPPH radical scavenging assay (Table 2), results of the present study revealed that the IC_50_ value of Xwak was 21.02 ± 0.33 *μ*g/mL. In ABTS radical scavenging assay (Table 3), IC_50_ value of Xwak was 14.39 ± 0.30 *μ*g/mL. Moreover, the IC_50_ value of the positive control on DPPH and ABTS was 5.18 ± 0.29 and 3.26 ± 0.36 *μ*g/mL, respectively. These results indicated that Xwak has a satisfactory antioxidant activity *in vitro.*

### 3.3. Effects of Xwak on Alcoholic Liver Injury

**Figure 1:**
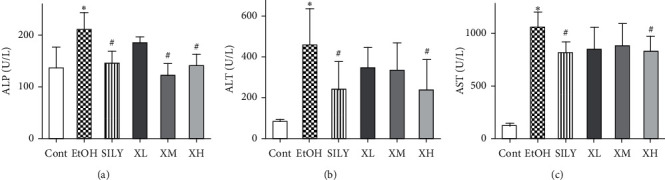
Effects of Xwak on the serum levels of alkaline phosphatase (ALP), alanine aminotransferase (ALT), and aspartate aminotransferase (AST). Data are expressed as the mean ± standard deviation (SD) (*n* = 8). ^*∗*^*p* < 0.05 compared with the Cont; ^#^*p* < 0.05 compared with the EtOH. Cont, control group; EtOH, model group; SILY, positive control; XL (low-dose of Xwak); XM, medium-dose of Xwak; XH, high-dose of Xwak.

The protective effects of Xwak on mice with alcoholic liver injury were evaluated using serum levels of AST, ALT, and ALP. As depicted in Figure 1, compared with the Cont group, the serum levels of AST, ALT, and ALP were significantly (*p* < 0.05) elevated in the EtOH group, indicating status of liver dysfunction in the EtOH group. The serum level of ALP was noticeably (*p* < 0.05) reduced in SILY, XM, and XH groups. The decreased serum levels of ALT and AST were also noted in the SILY group (*p* < 0.05) and XH group (*p* < 0.05). The abovementioned results indicated that Xwak could ameliorate the alcohol-induced liver injury in mice.

**Figure 2:**
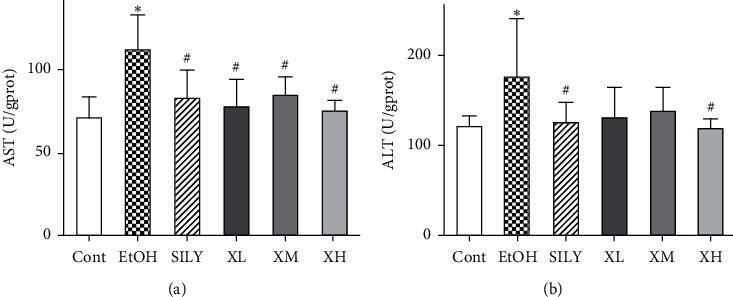
Effects of Xwak on aspartate aminotransferase (AST) and alanine aminotransferase (ALT) in the liver of mice. Values are expressed as the mean ± standard deviation (SD) (*n* = 8). ^*∗*^*p* < 0.05 compared with the Cont group; ^#^*p* < 0.05 compared with the EtOH group. Cont, control group; EtOH, model group; SILY, positive control; XL, low-dose of Xwak; XM, medium-dose of Xwak; XH, high-dose of Xwak.

The effects of Xwak on liver function were examined by measuring the levels of AST, ALT, NO, and LDH. As displayed in Figure 2, the levels of AST and ALT were markedly enhanced in the EtOH group (*p* < 0.05). It also was noted that the level of AST was significantly reduced by Xwak and silybin in SILY, XL, XM, and XH groups (*p* < 0.05). The level of ALT was also noticeably decreased in SILY and XH groups (*p* < 0.05).

**Figure 3:**
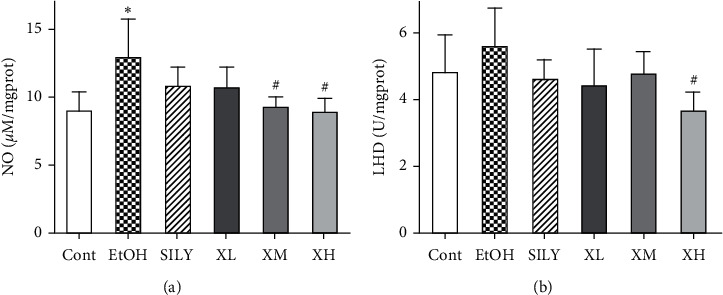
Effects of Xwak on the levels of nitric oxide (NO) and lactate dehydrogenase (LDH) in the liver of mice. Values are expressed as the mean ± standard deviation (SD) (*n* = 8). ^*∗*^*p* < 0.05 compared with the Cont group; ^#^*p* < 0.05 compared with the EtOH group. Cont, control group; EtOH, model group; SILY, positive control; XL, low-dose of Xwak; XM, medium-dose of Xwak; XH, high-dose of Xwak.

As shown in Figure 3, the level of NO was significantly increased in the EtOH group (*p* < 0.05) compared with that in the Cont group. The elevation of the level of NO could be attenuated by supplementation of Xwak, especially at XM and XH groups (*p* < 0.05). The level of LDH was increased in the EtOH group compared with that in the Cont group, while the difference was not statistically significant. The level of LDH was decreased by Xwak in the XH group (*p* < 0.05).

### 3.4. Effects of Xwak on Antioxidant Activities in Liver

**Figure 4:**
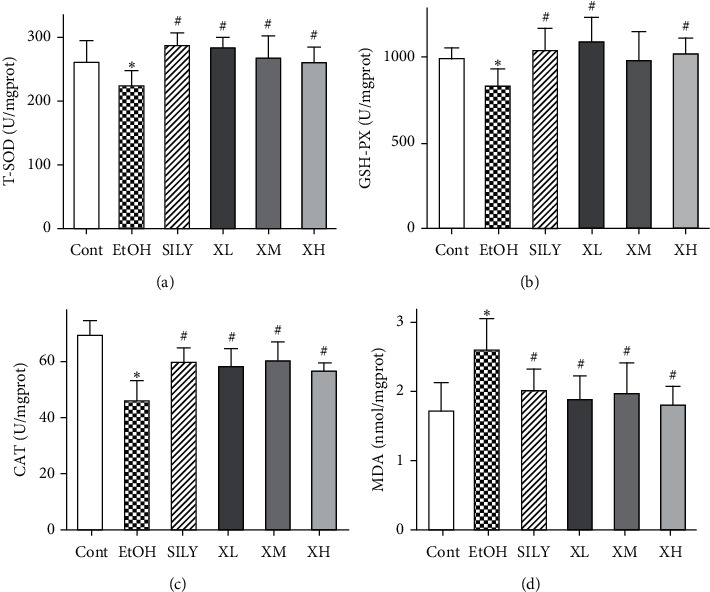
Effects of Xwak on the levels of total superoxide dismutase (T-SOD), glutathione peroxidase (GSH-PX), catalase (CAT), and malondialdehyde (MDA) in the liver of mice. Data are expressed as the mean ± standard deviation (SD) (*n* = 8). ^*∗*^*p* < 0.05 compared with the Cont group; ^#^*p* < 0.05 compared with the EtOH group. Cont, control group; EtOH, model group; SILY, positive control; XL, low-dose of Xwak; XM, medium-dose of Xwak; XH, high-dose of Xwak.

Several antioxidant enzymes were examined as parameters to detect the level of oxidative stress in the liver of mice (Figure 4). The levels of GSH-PX, T-SOD, and CAT were markedly reduced in the EtOH group (*p* < 0.05). However, the level of MDA was remarkably increased in the EtOH group (*p* < 0.05) compared with that in the Cont group. The levels of T-SOD and CAT were significantly increased in the three groups of XL, XM, and XH in mice with alcoholic liver injury (*p* < 0.05). The level of GSH-PX was notably increased in XL and XH groups (*p* < 0.05) compared with that in the EtOH group. Additionally, the level of MDA was noticeably decreased in the three groups of XL, XM, and XH (*p* < 0.05). These data demonstrated that Xwak has an acceptable antioxidant capacity *in vivo.*

### 3.5. Effects of Xwak on Lipid Levels in Liver

**Figure 5:**
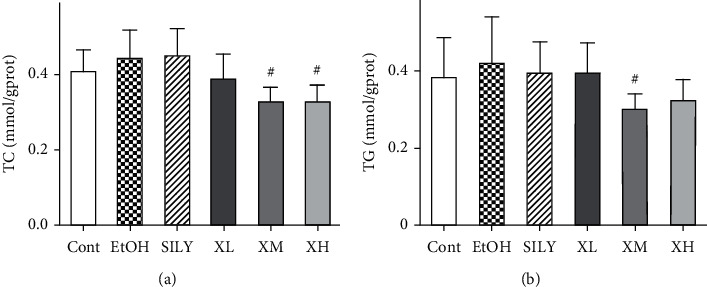
Effects of Xwak on the levels of total cholesterol (TC) and triglyceride (TG) in the liver of mice. Values are expressed as the mean ± standard deviation (SD) (*n* = 8). ^*∗*^*p* < 0.05 compared with the Cont group; ^#^*p* < 0.05 compared with the EtOH group. Cont, control group; EtOH, model group; SILY, positive control; XL, low-dose of Xwak; XM, medium-dose of Xwak; XH, high-dose of Xwak.

Herein, the effects of Xwak on the levels of TC and TG were measured in the liver of mice. As depicted in Figure 5, the levels of TC and TG were increased in the EtOH group compared with those in the Cont group, while the difference was not statistically significant. In mice that treated with Xwak, the level of TC was markedly reduced in the XM group and XH group (*p* < 0.05). A remarkable decrease of TG level was also noted in the XM group (*p* < 0.05). A trend of insignificant effect of Xwak on the levels of TC and TG was observed in other administration-based groups. These results indicated that Xwak may have a positive effect on hepatic lipid level.

### 3.6. Effects of Xwak on Levels of Cytokines in Liver

**Figure 6:**
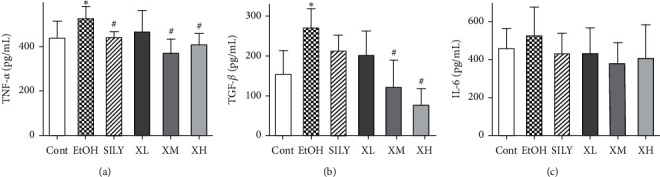
Effects of Xwak on the levels of tumor necrosis factor-*α* (TNF-*α*), transforming growth factor-*β* (TGF-*β*), and interleukin-6 (IL-6) in the liver of mice. Values are expressed as the mean ± standard deviation (SD) (*n* = 8). ^*∗*^*p* < 0.05 compared with the Cont group; ^#^*p* < 0.05 compared with the EtOH group. Cont, control group; EtOH, model group; SILY, positive control; XL, low-dose of Xwak; XM, medium-dose of Xwak; XH, high-dose of Xwak.

As shown in Figure 6, the levels of TNF-*α* and TGF-*β* in the EtOH group were significantly elevated compared with those in the Cont group (*p* < 0.05). However, the levels of TNF-*α* and TGF-*β* were significantly reduced in the XM and XH groups (*p* < 0.05). The level of IL-6 in the EtOH group was higher than that in the Cont group, whereas the difference was not statistically significant. The increased level of IL-6 in mice with alcoholic liver injury was attenuated after treatment with silybin and Xwak.

### 3.7. Effects of Xwak on the Expression Levels of ERK, P38MAPK, NF-*κ*B, iNOS, COX-2, CYP2E1, Nrf, and HO-1 in the Liver of Mice

**Figure 7:**
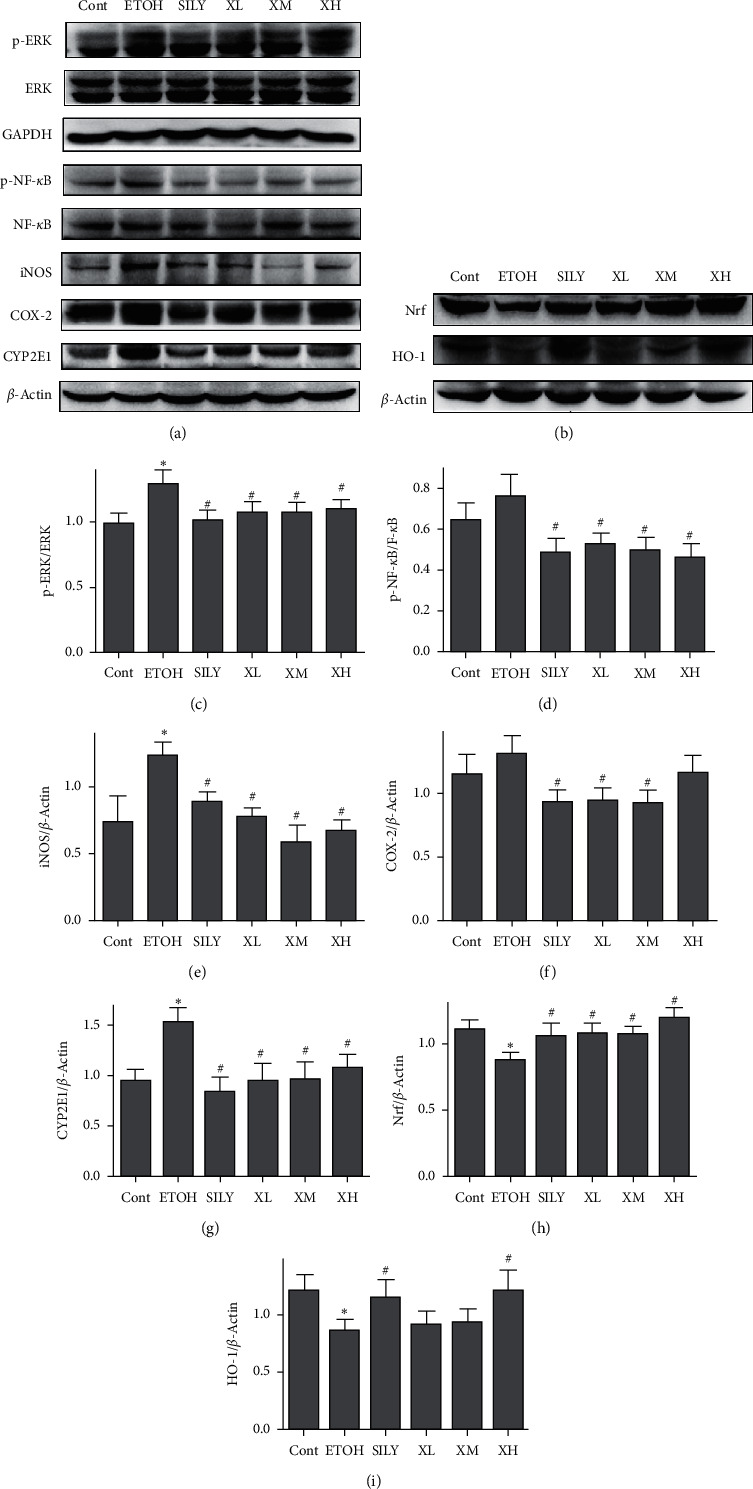
Effects of Xwak on protein expression level were measured by Western blot analysis. (a) The bands of ERK/NF-*κ*B signaling pathways. (b) The bands of Nrf/HO-1 signaling pathways. Quantification of different protein expressions. (c) Quantification of p-ERK/ERK, (d) quantification of p-NF-*κ*B/NF-*κ*B, (e) quantification of iNOS/*β*-actin, (f) quantification of COX-2/*β*-actin, (g) quantification of CYP2E1/*β*-actin, (h) quantification of Nrf2/*β*-actin, and (i) quantification of HO-1/*β*-actin. Values are expressed as the mean ± standard deviation (SD). Cont, control group; EtOH, model group; SILY, positive control; XL, low-dose of Xwak; XM, medium-dose of Xwak; XH, high-dose of Xwak.

We further explored the underlying hepatoprotective mechanism of Xwak in mice with alcoholic liver injury. The results of Western blotting showed that the expression levels of p-ERK1/2, p-NF-*κ*B, COX-2, iNOS, and CYP2E1 were elevated in the EtOH group compared with those in the Cont group (see Figure 7). The increased expression levels of p-ERK1/2, p-NF-*κ*B, COX-2, iNOS, and CYP2E1 were significantly attenuated after treatment with silybin and Xwak. Moreover, the expression levels of Nrf and HO-1 were noticeably reduced in the EtOH group compared with those in the Cont group. The reduced expression level of Nrf was significantly improved after treatment with silybin and Xwak in all groups (SILY, XL, XM, and XH groups). The expression level of HO-1 was also improved in SILY and XH groups. Our results indicated that Xwak is able to decrease activities of ERK and improve activities of Nrf/HO-1 signaling pathways in mice with alcoholic liver injury.

### 3.8. Effects of Xwak on Liver Histopathology

**Figure 8:**
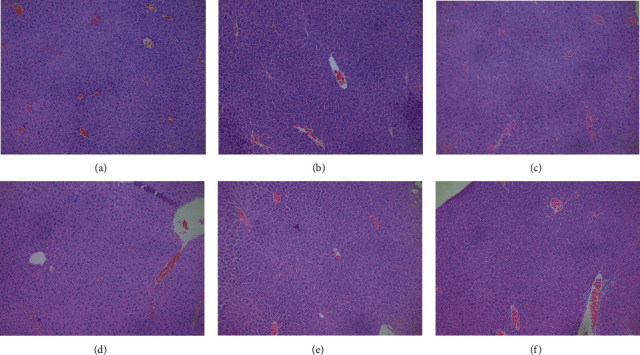
Effects of Xwak on liver histopathology. (a) Cont (control group), (b) EtOH (model group), (c) SILY (positive control), (d) XL (low-dose of Xwak), (e) XM (medium-dose of Xwak), and (f) XH (high-dose of Xwak).

No obvious pathological changes were found in the pathological sections of liver tissue in each group (see Figure 8).

## 4. Discussion

Oxidative stress plays a significant role in the pathogenesis of alcoholic liver injury. The metabolism of ethanol to acetaldehyde and then to acetate is associated with the production of reactive oxygen species (ROS) accentuating the oxidative state of cells [32, 33]. In the present study, we evaluated the scavenging effects of Xwak on free radicals of ABTS and DPPH *in vitro*, and the results showed that Xwak has a satisfactory antioxidant activity and can be used as a potential free radical scavenger. Therefore, we further studied the antioxidant and anti-inflammatory activities of Xwak in the mouse model of alcoholic liver injury, in order to clarify its hepatoprotective mechanism.

The alanine aminotransferase (ALT) and aspartate aminotransferase (AST) are both found in serum, in the liver, and in various organ tissues. Transaminases are rapidly released into blood stream when injuries or diseases affected the body tissues [34]. The results of the present study unveiled that the levels of AST, ALT, and ALP in the mouse model of acute alcoholic liver injury were significantly increased in serum, which is consistent with findings of previous reports [35]. This indicated that the mouse model of acute alcoholic liver injury was successfully established. Furthermore, we measured the increased levels of AST and ALT in the liver, but they are not as significant as they are in serum, and these results showed that acute alcoholic liver injury can cause a rapidly increased transaminase levels in serum, while the levels of transaminase in the liver were increased slowly, but both serum and liver transaminase levels were improved by Xwak extract treatment. Moreover, the increased levels of NO and LDH in the liver of mice were also reduced by Xwak. These data suggested that Xwak could improve liver function in mice with alcoholic liver injury.

Ethanol treatment leads to the production of a great number of ROS and then enhances oxidative stress in body tissues. A variety of enzymatic and nonenzymatic systems participate in protecting cells from ROS, such as CAT, GSH-PX, and T-SOD [36]. The levels of CAT, GSH-PX, and T-SOD were measured in the mouse model of alcoholic liver injury. The results showed that the activities of these three enzymes were significantly reduced in the mouse model of alcoholic liver injury. Treatment with Xwak noticeably improved the activities of CAT, GSH-PX, and T-SOD. MDA is one of the most known secondary products of lipid peroxidation, and it can be used as a marker of cell membrane injury. The results of the current research uncovered that the level of MDA was significantly reduced after treatment with Xwak. The abovementioned results showed that Xwak could alleviate the oxidative stress-induced liver injury through antioxidant activity.

Hepatoprotective cytokines, e.g., IL-6, are associated with ALD [37]. The production of TNF-*α* is one of the earliest events in several types of liver injuries [38]. In addition, TNF-*α* can induce other cytokines, such as IL-6, and recruit more inflammatory cells to damage the hepatocytes. TGF-*β* is a multifunctional cytokine involved in various pathologic conditions, including carcinogenesis and tissue fibrosis [39]. We, in the present research, measured the levels of three important cytokines (TNF-*α*, TGF-*β*, and IL-6). It was revealed that the levels of TNF-*α* and TGF-*β* were increased in the mouse model of alcoholic liver injury, which accompanied with inflammation-mediated disruption. However, the levels of pro-inflammatory cytokines were improved in the mouse model of alcoholic liver injury after treatment with Xwak. The improvement of inflammation-mediated disruption may conducive to the Xwak activity in the control of liver injury.

ERKs are widely expressed protein kinase intracellular signaling molecules, and a number of studies demonstrated that ERK plays a substantial role in liver diseases [40–42]. The association between ERK and NF-*κ*B pathways in response to inflammatory signals regulates several inflammatory factors, such as TNF-*α*, NO, iNOS, and COX-2 [43–45]. Treatment with Xwak amended the high expression levels of p-ERK, p-NF-*κ*B, iNOS, and COX-2 in the mouse model of alcoholic liver injury. The activation of the ERK/NF-*κ*B signal pathway was accompanied with the improvement of some inflammatory factors, and the levels of TNF-*α*, TGF-*β*, IL-6, and NO were also ameliorated in the mouse model of alcoholic liver injury after treatment with Xwak.

Anti-inflammatory and antioxidants agents are often considered beneficial in the treatment of liver diseases. The main compounds of Xwak are chlorogenic acid and flavonoids, and these compounds were found to have the effect of controlling oxidative and inflammatory stress conditions, which is also consistent with our results [10, 46]. CYP2E1 is one of the major ROS generators in the liver and is recognized as a risk factor for alcoholic liver disease [47–49]. Some studies previously indicated that the Nrf2/HO-1 signaling pathway is involved in the mechanism of antioxidation, anti-inflammation, and cell protection [50–53]. The results of the current research showed that the expression level of CYP2E1 was inhibited, and the Nrf2/HO-1 signaling pathway was activated in the mouse model of alcoholic liver injury after treatment with Xwak. Moreover, the activity of antioxidant enzyme improved. The state of oxidative stress in the mouse model of alcoholic liver injury was alleviated by Xwak.

The change of biochemical indexes in liver function is that the body has started the compensation mechanism (self-protection), which can protect the body from damage when the external low-intensity stimulation. In the case of functional decompensation, cell damage will be shown and there will be obvious changes in morphology. Since we used the model of acute liver injury induced by intraperitoneal injection, the animals were killed 5 hours after injection, and the relevant indexes were detected. Therefore, there was no obvious morphological change in the liver, so no obvious histopathological changes were observed in the section.

Based on the experimental results, the medium-dose and high-dose of Xwak have shown the hepatoprotective effect in acute alcoholic liver injury mice and similar to silybin potency. The dose of Xwak administered is larger than silybin, which is calculated based on the specifications of clinical medications. Therefore, compared with the positive drug silybin, as a classic folk prescription, Xwak has a certain hepatoprotective effect in acute alcoholic liver injury mice. Furthermore, some of the detection indexes in this study will be carried out in the follow-up experiments.

In conclusion, our findings showed hepatoprotective influence of Xwak through activation of ERK/NF-*κ*B and Nrf2/HO-1 signaling pathways to regulate the redox balance and reduce the oxidative stress-induced inflammatory response. This study may provide a reliable scientific basis for the clinical application of Xwak.

## Figures and Tables

**Table 1:** Identification of extracts from Xwak using UHPLC-Q-Orbitrap MS.

No.	Retention time (min)	Exact mass (*m/z*)	Molecular formula	Error (ppm)	MS^2^ data (*m/z*)	Identification
1	2.74	195.05034	C_6_H_12_O_7_	2.10	129(20), 75(100)	Gluconic acid
2	2.89	341.10883	C_12_H_22_O_11_	2.89	179(5), 113(20), 89(40), 71(70), 59(100)	Caffeic acid-glucoside
3	2.92	191.05537	C_7_H_12_O_6_	1.88	93(20), 85(100)	Quinic acid
4	3.36	133.01324	C_4_H_6_O_5_	0.67	115(80), 71(100)	Malic acid
5	4.47	191.01901	C_6_H_8_O_7_	2.01	129(10), 111(100)	Critic acid
6	5.41	343.06739	C_14_H_16_O_10_	4.29	191(80), 169(70), 125(95), 107(100)	Galloyle-quinic acid
7	5.87	481.06250	C_20_H_17_O_14_	2.54	301(100), 275(60), 257(30), 229(40), 185(20)	HHDP-glucoside
8	5.91	331.0674	C_13_H_16_O_10_	3.93	169(80), 125(100)	Galloyle-glucoside
9	8.78	169.01338	C_7_H_6_O_5_	1.36	125(100)	Gallic acid
10	9.21	343.10367	C_14_H_16_O_10_	3.49	191(100), 169(70), 125(85), 107(90)	Galloyle-quinic acid
11	9.29	483.07867	C_20_H_20_O_14_	3.60	331(10), 169(60), 125(100), 107(30)	Digalloyl-glucoside
12	12.05	483.07825	C_20_H_20_O_14_	2.73	331(10), 169(60), 125(100), 107(20)	Digalloyl-glucoside
13	13.63	483.07828	C_20_H_20_O_14_	2.79	331(15), 169(60), 125(100), 107(20)	Digalloyl-glucoside
14	15.44	153.01847	C_7_H_6_O_4_	1.52	109(100)	2,3-Dihydroxybenzoic acid
15	17.98	353.08796	C_16_H_18_O_9_	3.53	191(80), 179(30), 161(10), 135(100), 85(30)	5-O-caffeoylquinic acid
17	18.01	483.07819	C_20_H_20_O_14_	2.31	313(25), 169(80), 125(100), 107(30)	Digalloyl-glucoside
18	22.76	339.07232	C_15_H_16_O_9_	3.72	177(100), 133(30), 105(20), 89(20)	Esculin hydrate
19	35.40	177.01860	C_9_H_6_O_4_	2.06	149(20), 133(90), 105(50), 89(40)	Esculetin
20	35.94	353.08795	C_16_H_18_O_9_	3.47	191(100), 161(5), 127(5), 93(15), 85(30)	3-O-caffeoyl quinic acid
21	38.64	353.08807	C_16_H_18_O_9_	3.86	191(40), 179(30), 173(50), 161(5), 135(100), 127(5), 93(50)	4-O-caffeoyl quinic acid
22	64.99	367.1045	C_17_H_20_O_9_	2.97	191(100), 193(20), 173(10), 155(5), 134(60), 93(80)	Feruloylquinic acid
23	65.23	515.11951	C_25_H_24_O_12_	1.97	353(10), 191(95), 179(50), 161(15), 135(100), 85 (20)	1,3-Di-O-caffeoyl quinic acid
24	65.24	635.08911	C_27_H_24_O_18_	1.92	465(50), 313,(40), 169(95), 125(100), 107(20)	Tri-O-galloyl-glucoside
25	66.52	469.0049	C_21_H_30_O_15_	1.80	300(100), 271(20), 229(30), 185(10)	Ellagic acid blactone
26	97.66	300.99887	C_14_H_6_O_8_	3.24	284(10), 245(20), 229(30), 185(10)	Ellagic acid
27	98.11	463.08832	C_21_H_20_O_12_	2.41	301(100)175(20), 151(50), 107(30)	Quercetin-7-O-glucoside
28	102.55	463.08832	C_21_H_20_O_12_	2.63	300(70), 271(100), 255(50), 243(40), 227(20), 151(20)	Hyperoside
29	103.47	609.14618	C_27_H_30_O_16_	1.92	300(90), 271(100), 255(50), 243(30), 227(10), 151(20)	Rutin
30	106.13	447.0934	C_21_H_20_O_11_	2.89	285(100), 256(20), 227(20), 133(30)	Luteolin-O-glucoside
31	106.41	477.06766	C_21_H_18_O_13_	2.71	301(100), 255(30), 227(10) , 179(35), 151(60), 107(30)	Quercetin-3-O-glucuronide
32	107.45	463.08844	C_21_H_20_O_12_	2.89	300(90), 271(100), 255(50), 243 (30), 227(20), 151(10)	Quercetin-3-O-glucoside
33	107.37	681.13068	C_29_H_30_O_16_	1.36	351(40), 299(60), 193(40), 113(80)	Tricin-7-O-diglucuronide
34	108.05	463.08847	C_21_H_20_O_12_	2.95	300(80), 271(100), 255(50), 243(30), 227(20), 151(15)	Quercetin-3′-O-glucoside
35	115.70	433.07784	C_20_H_18_O_11_		300(90), 271(100), 255(50), 243(30), 227(20), 151(15)	Quercetin-3-O-arabinoside
36	116.39	515.11957	C_25_H_24_O_12_	2.27	353(10), 191(50), 179(50), 173(55), 161(25), 135(100), 93(40)	3,4-Di-O-caffeoyl quinic acid
37	120.79	515.11963	C_25_H_24_O_12_	2.38	353(10), 191(50), 179(50), 173(55), 161(25), 135(100)	3,5-Di-O-caffeoyl quinic acid
38	129.63	447.09344	C_21_H_20_O_11_	2.80	284(50), 255(90), 227(100), 183(20), 151(5)	Kampferol-O-glucoside
39	130.18	447.09344	C_21_H_20_O_11_	2.81	300(85), 271(100), 255(55), 243(30), 227(15), 179(10), 151(20)	Quercetin-3-O-rhahmanoside
40	136.16	515.11938	C_25_H_24_O_12_	1.90	353(20), 191(40), 179(45), 173(70), 161(20), 135(100)	4,5-Di-O-caffeoyl quinic acid
41	140.94	431.09863	C_21_H_20_O_10_	3.15	284(50), 255(100), 227(90), 211(20), 183(10)	Kampferol-O-rhamnoside
42	141.43	417.11948	C_21_H_22_O_9_	3.51	255(100), 180(80), 153(30), 148(70), 135(80), 119(95), 108(50), 91(100)	Isoliquiritigenin-O-glucoside
43	145.43	529.13531	C_26_H_26_O_12_	2.38	367(5), 193(20), 179(5), 173(100), 161(20), 134(50), 93(70)	4-O-feruloyl, 5-O-caffeoy quinic acid
44	149.76	285.04050	C_15_H_10_O_6_	3.98	257(5), 243(5), 217(5), 131(100)	Luteolin
45	150.45	253.05049	C_15_H_10_O_4_	3.77	225(100), 210(10), 181(5)	Daidzein
46	150.53	431.09839	C_21_H_20_O_10_	2.59	269(100), 240(10), 225(40), 181(10)	Apigenin-O-gucoside
47	155.98	247.13373	C_15_H_20_O_3_	3.47	231(5), 203(100), 188(10), 163(20), 109(30)	Rupestonic acid
48	157.41	299.05627	C_16_H_12_O_6_	4.21	284(100), 256(80), 151(10)	Diosmetin

**Table 2:** DPPH radical scavenging assay of Xwak (*n* = 3).

Sample	Concentration (*μ*g/mL)	Inhibition rate (%)	IC_50_ (*μ*g/mL)
Xwak	156.25	93.01	21.02 ± 0.33
	78.13	91.66	
	39.06	74.24	
	19.53	47.38	
	9.76	21.26	

Vitamin C	25	93.74	5.18 ± 0.29
	12.5	86.16	
	6.25	57.23	
	3.125	27.51	
	1.5625	12.56	

**Table 3:** ABTS radical scavenging assay of Xwak (*n* = 3).

Sample	Concentration (*μ*g/mL)	Inhibition rate (%)	IC_50_ (*μ*g/mL)
Xwak	50	99.75	14.39 ± 0.30
	25	64.24	
	12.5	35.28	
	6.25	18.92	
	3.13	11.25	

Vitamin C	8	97.79	3.26 ± 0.36
	6	74.80	
	4	49.12	
	2	23.40	
	1	11.41	

## Data Availability

Derived data supporting the findings of this study are available from the corresponding author upon a reasonable request.

## Supplementary Materials

Supplementary Materials LC-MS analysis of Xwak.
