# Supplementary material for: Chemical Components and Hepatoprotective Mechanism of Xwak Granule in Mice Treated with Acute Alcohol
Source: Evid Based Complement Alternat Med. 2020 Sep 28;2020:8538474. doi: 10.1155/2020/8538474 (PMC7539125; doi:10.1155/2020/8538474)
Supplement: Supplementary Materials — LC-MS analysis of Xwak. [file 8538474.f1.pdf]

LC-MS Analysis of Xwak

Flavonoids

Analysis of compounds **27** (t_R = 98.11 min), **28** (t_R = 102.55 min), **29** (t_R = 103.47 min), **31** (t_R = 106.41 min), **32** (t_R = 107.45 min), **34** (t_R = 108.05 min) , **35** (t_R = 115.70 min) and **39** (t_R = 130.18 min) showed $[M-H]^-$ ions at *m/z* 463, 463, 609, 477, 463, 463, 433, and 447, respectively. In the MS^2 experiment (Table 1), these compounds have the same characteristic fragment ions at *m/z* 301, 271, 255, 243, 227 and 151, indicating that these compounds have the same quercetin aglycone. Therefore, compounds **27**, **28**, **29**, **31**, **32**, **34**, **35,** and **39** were identified as quercetin-7-O-glucoside (quercimeritrin), hyperoside, rutin, quercetin-3-O-glucuronide, isoquercetrin, quercetin-3′-O-glucoside (Quercitrin), quercetin-3-O- arabinoside, and astralagin, respectively.

In addition, other flavonoids were detected according to the above-mentioned methods. Compounds **30** (t_R = 106.13 min) and **44** (t_R = 149.76 min) were tentatively identified as luteolin-O-glucoside and luteolin, respectively. Compounds **38** (t_R = 129.63 min) and **41** (t_R = 140.94 min) were tentatively assumed as kampferol-O-glucoside and kampferol-O-rhahmanoside, respectively. Compound **33** (t_R = 107.37 min) was confirmed as tricin-7-O-diglucuronide. Compound **42** (t_R = 141.43 min) was tentatively detected as liquiritigenin-O-glucoside or isoliquiritigenin-O-glucoside. Compounds **45** (t_R = 150.45 min), **46** (t_R = 150.53 min), and **48** (t_R = 157.41 min) were confirmed as daidzein, apigenin-7-O-glucoside, and diosmetin, respectively.

Tannins

Compounds **8** (t_R = 5.91 min), **11** (t_R = 9.21 min), **12** (t_R = 12.05 min), and **13** (t_R = 13.63 min) produced the same characteristic ions of gallic acid at *m/z* 169 and 125 (Table 1). For compound **8**, $[M-H]^-$ ions at *m/z* 331 showed loss of glucoside. Therefore, compound **8** was tentatively assumed as Galloyle-glucoside. For

compounds **11**, **12**, and **13**, they had the same $[M-H]^-$ ions at *m/z* 483, and had the same fragment ions at *m/z* 331 [M-H-glucoside], indicating that these three compounds were isomers. According to a previous study [1], compounds **11**, **12**, and **13** were tentatively identified as Digalloyl-glucoside and isomers.

According to the above-mentioned inference methods, compounds **2** (t_R = 2.89 min) and **7** (t_R = 5.87 min) were tentatively identified as caffeic acid 3-glucoside and HHDP-glucoside, respectively. Compounds **6** (t_R = 5.41 min) and **10** (t_R = 9.21 min) were found as Galloyle-quinic acid and isomers, respectively.

Chlorogenic acids

Herein, 9 chlorogenic acids were detected, including caffeoylquinic acid (CQA), feruloylquinic acid (FQA), feruloylcaffeoylquinic acid (FCQA), dicaffeoylquinic acid (diCQA), and their deriveters (Table 1).

Compounds **15** (t_R = 17.98 min), **20** (t_R = 35.95 min), and **21** (t_R = 38.64 min) displayed the same $[M-H]^-$ ions at *m/z* 353. In the MS^2 spectra (Table 1), they had fragment ions at *m/z* 191 [quinic acid-H]$^-$, *m/z* 179 [caffeic acid-H]$^-$, 173 [quinic acid-H_2O-H]$^-$, 161 [caffeic acid-H_2O-H]$^-$, and 135 [caffeic acid-H_2O-CO_2-H]$^-$, indicating that these compounds had quinic acid and caffeic acid in their structure. Therefore, we compared the retention time and fragmentation behavior with the reference standards, and compounds **15, 20,** and **21** were confirmed as 5-O-Caffeoylquinic acid, 3-O-Caffeoylquinic acid, and 4-O-caffeoylquinic acid, respectively. Compound **22** (t_R = 64.99 min) was tentatively identified as 5-O-feruloylquinic acid. Compounds **23** (t_R = 65.23 min), **36** (t_R = 116.39 min), **37** (t_R = 120.79 min), and **40** (t_R = 136.16 min) were detected as 1,3-di-*O*-caffeoyl quinic acid, 3,4-di-*O*-caffeoyl quinic acid, 3,5-di-*O*-caffeoyl quinic acid, and 4,5-di-*O*-caffeoyl quinic acid, respectively. Compound **43** (t_R = 145.43 min) was tentatively identified as 4-O-feruloyl,5-O-caffeoy quinic acid.

Phenolic acids and other compounds

Compounds **1** (t_R = 2.74 min), **3** (t_R = 2.92 min), **4** (t_R = 3.36 min), **5** (t_R = 4.47

min), **9** (t_R = 8.78 min), **14** (t_R = 15.44 min), and **26** (t_R = 97.66 min) displayed $[M-H]^-$ ions at *m/z* 195, 191, 133, 191, 169, 153, and 301, respectively. With comparing retention time and fragmentation patterns with reference standards, compounds **1**, **3**, **4**, **5**, **9**, **14,** and **26** were confirmed as gluconic acid, quinic acid, malic acid, critic acid, gallic acid, 2,3-dihydroxybenzoic acid, and ellagic acid, respectively. Compounds **18** (t_R = 22.76 min), **19** (t_R = 35.40 min), and **47** (t_R = 115.98 min) were confirmed as Esculin hydrate, Esculetin, and rupestonic acid, respectively.

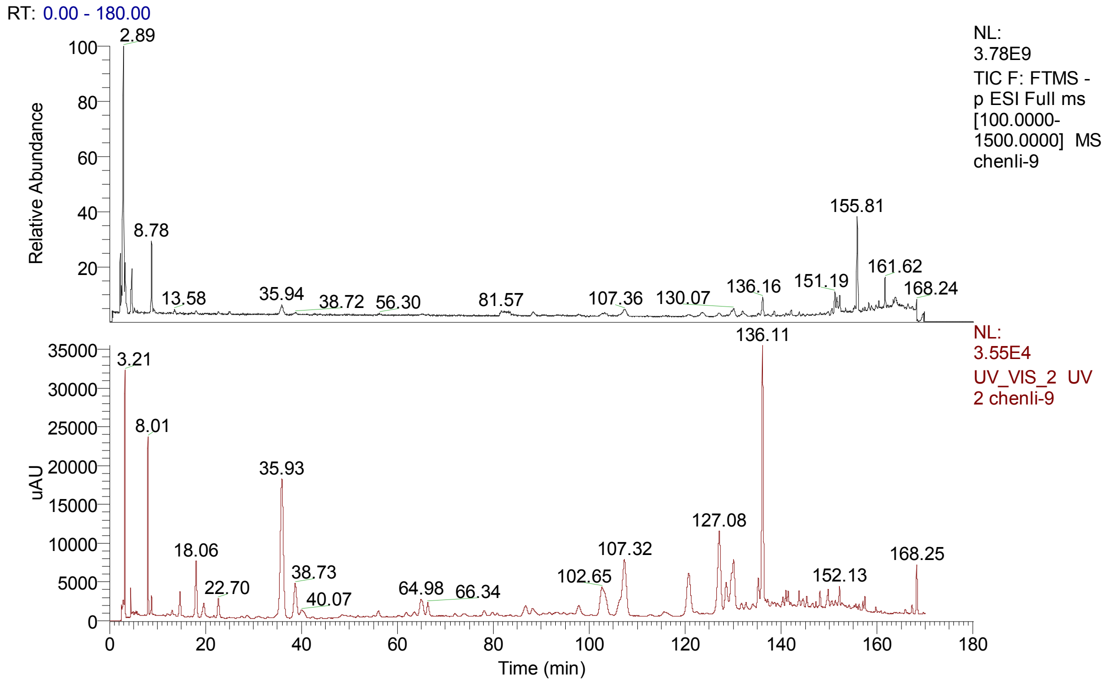

LC-MS Chromatogram of Xwak

Reference

1. R. Abdulla, S. Mansur, H. Lai, et al. "Qualitative Analysis of Polyphenols in Macroporous Resin Pretreated Pomegranate Husk Extract by HPLC-QTOF-MS," Phytochem Anal, vol. 28, no. 5, pp. 465-473.
